# Distinguishing Laparoscopic Surgery Experts from Novices Using EEG Topographic Features

**DOI:** 10.3390/brainsci13121706

**Published:** 2023-12-11

**Authors:** Takahiro Manabe, F.N.U. Rahul, Yaoyu Fu, Xavier Intes, Steven D. Schwaitzberg, Suvranu De, Lora Cavuoto, Anirban Dutta

**Affiliations:** 1 School of Engineering, University of Lincoln, Lincoln LN6 7TS, UK; tmanabe@u.northwestern.edu; 2 Centre for Modeling, Simulation, and Imaging in Medicine, Rensselaer Polytechnic Institute, Troy, MI 12180, USA; rahul@rpi.edu (F.R.); intesx@rpi.edu (X.I.); 3 Department of Industrial and Systems Engineering, University at Buffalo, Buffalo, NY 14260, USA; yaoyufu@buffalo.edu (Y.F.); loracavu@buffalo.edu (L.C.); 4 Department of Biomedical Engineering, Rensselaer Polytechnic Institute, Troy, MI 12180, USA; 5 School of Medicine and Biomedical Sciences, University at Buffalo, Buffalo, NY 14203, USA; schwaitz@buffalo.edu; 6 College of Engineering, Florida A&M University-Florida State University, Tallahassee, FL 32310, USA; sde@eng.famu.fsu.edu

**Keywords:** fundamentals of laparoscopic surgery, electroencephalogram, skill classification, common spatial pattern, temporal–spatial pattern recognition, deep neural networks

## Abstract

The study aimed to differentiate experts from novices in laparoscopic surgery tasks using electroencephalogram (EEG) topographic features. A microstate-based common spatial pattern (CSP) analysis with linear discriminant analysis (LDA) was compared to a topography-preserving convolutional neural network (CNN) approach. Expert surgeons (N = 10) and novice medical residents (N = 13) performed laparoscopic suturing tasks, and EEG data from 8 experts and 13 novices were analysed. Microstate-based CSP with LDA revealed distinct spatial patterns in the frontal and parietal cortices for experts, while novices showed frontal cortex involvement. The 3D CNN model (ESNet) demonstrated a superior classification performance (accuracy > 98%, sensitivity 99.30%, specificity 99.70%, F1 score 98.51%, MCC 97.56%) compared to the microstate based CSP analysis with LDA (accuracy ~90%). Combining spatial and temporal information in the 3D CNN model enhanced classifier accuracy and highlighted the importance of the parietal–temporal–occipital association region in differentiating experts and novices.

## 1. Introduction

Laparoscopic surgery training is a comprehensive module under the Fundamentals of Laparoscopic Surgery (FLS) curriculum, aimed at equipping medical professionals, scientists, and doctors with basic surgical skills required for successful laparoscopic procedures. FLS is a joint program by the American Gastrointestinal and Surgery Association and the American Academy of Surgery for general surgery [1]. FLS certification involves five psychomotor tasks of increasing complexity: pegboard transfer, pattern cutting, placement of a ligating loop, suturing with extracorporeal knot tying, and suturing with intracorporeal knot tying. This training focuses on cognitive and psychological abilities essential for minimally invasive surgery and serves as a standardised assessment of physicians’ capabilities where brain correlates are important to robustly identify expertise [2].

To evaluate brain correlates of FLS skills, it is proposed that the brain forms a cognitive-perceptual mental model [3,4] during the initial stages of the skill acquisition in a novel laparoscopy environment [5,6,7,8]. Here, Fitts and Posner proposed a three-stage model for motor skill acquisition, comprising the cognitive stage, the associative stage, and the autonomous stage [9]. During these stages for motor skill acquisition, the brain–behaviour relationship can be explored based on portable brain imaging. Brain circuit mechanisms, driven by motor skill proficiency, involve selective attention and cortical alterations in motor planning and execution [6]. For example, specific brain mechanisms [10] subserved by motor skill proficiency may represent dissociable selective attention or local excitability alterations in the cortex during motor planning and execution that are postulated to be driven by a supplementary motor area, premotor cortex, and cerebellum [11]—all communicating via the thalamus [12] and the corticothalamic loops [13,14]. In this study, we hypothesised that these semi-stable brain states involving selective attention and cortical alterations in motor planning and execution can be estimated based on the topography of electroencephalogram (EEG). The majority of contemporary brain–computer interfaces utilizing EEG rely on machine learning algorithms [15] and a wide array of classifier types is employed within this domain including facial expressions as control commands [16].

In this study, it is postulated that while experts will already have a cognitive perceptual model for FLS task performance based on their prior experience, the novices will start building the cognitive perceptual model on their first exposure to the FLS task [3,4]. Semi-stable brain states have been analysed in prior studies based on microstates [17], which can be estimated based on the scalp potential field or EEG topography [18], and are differentially modulated by the vigilance level [19]. Microstates approach to analyse brain states has an a priori assumption that only one spatial map accurately defines the brain’s global state at a given time and that the residuals are considered noise [18]. This microstate-based analysis has been applied to error-based learning using EEG in conjunction with functional near-infrared spectroscopy during a complex surgical motor task [20]. Based on related prior studies [14,21], we postulated in this study that the EEG topography during FLS suturing with intracorporal knot tying task will differ between experts and novices. First, we applied conventional common spatial pattern (CSP) approach, one of the most common methods for feature extraction in brain–computer interfaces [22], to classify two skill levels, experts and novices. We improved the traditional CSP method that is known to suffer from noise sensitivity due to the L2 norm in its optimisation problem to find a spatial filter [23]. We developed a microstate-based CSP approach that performed better due to a metacriterion that favours the highest signal-to-noise ratio [24]. We presented these preliminary results using microstate-based CSP approach at the Interservice/Industry Training, Simulation, and Education Conference (I/ITSEC) 2022 [21]. In our I/ITSEC presentation, we focused on a cohort of 10 expert surgeons and 10 novice medical residents. Through the application of a linear discriminant analysis with 10-fold cross-validation, we successfully attained a classification accuracy exceeding 90%, utilizing spatial pattern vectors extracted from the scalp. In this paper, we present a more advanced convolutional neural network (CNN) based approach with Grad-CAM analysis [25] and compare the results with our microstate-based CSP approach.

Our current study is motivated by prior studies that show non-Markovian and nonstationary microstates [26] can classify cognitive states using the attention-based time series deep learning framework [27]. However, the challenge remains in pre-selecting scalp potential topographies (microstates) that are considered stable [18], while short periods of unstable EEG topographies may occur, e.g., during errors. Instead of preselecting EEG microstates, one can use EEG topography as 3D tensor input for attention features in a CNN, e.g., see ESNet [28], where topography-preserving EEG-based temporal attentive pooling may be neurophysiologically interpretable [29] using Grad-CAM analysis [25]. Therefore, we adapted ESNet [28] to classify experts versus novices and compared the temporally important topography-preserving time segments with the microstate-based CSP approach [21] for mechanistic insights into skill learning [30]. Mechanistic insights were guided by prior studies [30,31] using functional near-infrared spectroscopy (fNIRS) that found cortical activation in the right prefrontal cortex, the right precentral gyrus, and the right postcentral gyrus at the start of the FLS task. Then, the left inferior frontal gyrus, the optical part, the left superior frontal gyrus, the medial orbital, the left postcentral gyrus, the gyrus, the left superior temporal gyrus, right superior frontal gyrus, and the medial orbital cortical areas of the cortical areas of the orbital showed significant differences between experts and novices in the error epochs [20]. In the current study, we analysed a subset of participants from prior studies [20,30] where simultaneous EEG measurements were conducted for our EEG topographic feature analysis. 

## 2. Methods

### 2.1. Subjects and Experimental Setup

The study received approval from the Institutional Review Board of the University at Buffalo, USA, and all procedures adhered to local research regulations for human subjects. Thirteen neurologically normal novice medical students (seven females) and ten experienced surgeons (five females), all right-handed, participated after providing written informed consent. The EEG data utilised in this study are a subset of a previously published study [20,30] where multimodal fNIRS-EEG data were collected.

The experienced surgeons, with 1 to 25 years of expertise in FLS laparoscopic suturing tasks, were compared to novices new to FLS suturing with intracorporal knot tying. A prior study [30] demonstrated a statistically significant superior performance of experienced surgeons (overall score: 370.4, SD: 61.3) compared to novices (overall score: 84.2, SD: 65.3). Participants received verbal instructions and were equipped with laparoscopic tools for the task, which involved suturing through two marks in a Penrose drain and tying specific knots using needle drivers operated by both hands. The task began with the ‘start’ command, recorded in the multimodal data, and concluded when the subject cut both ends of the suture within a 180 s timeframe.

**Figure 1:**
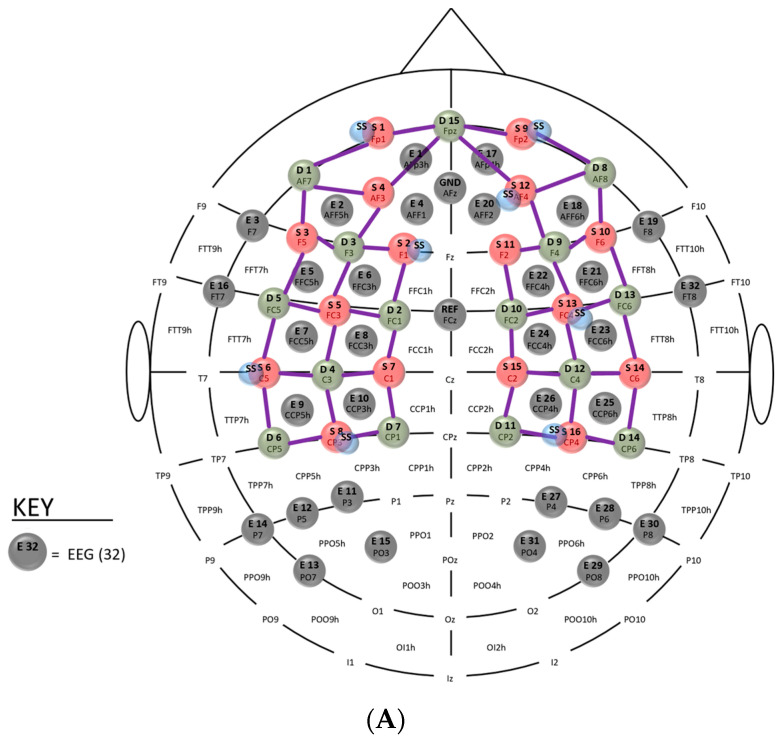

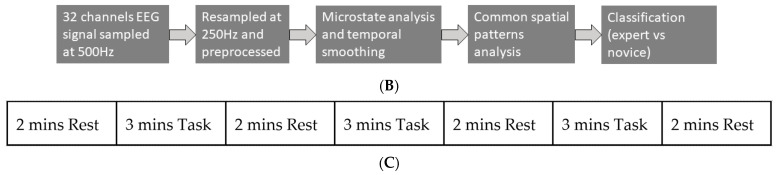
(**A**) Multimodal sensor montage where the 32 active EEG gel electrodes (E1–E32) are shown with grey discs. The fNIRS source (S1–S16) and detectors (D1–D15) were not used in the current study. (**B**) EEG data processing pipeline in EEGLab and BCILab for the CSP-based classification of experts versus novices. (**C**) Repeated measures of FLS task (3 min) with rest periods (2 min).

A customised multimodal fNIRS-EEG montage with 32 active gel electrodes (Figure 1A) was used to record brain activation signals. The EEG signals were captured by a wireless LiveAmp system (Brain Vision, Brain Vision, LLC-515 N. Greenfield Parkway, Suite 100, Garner, NC 27529, USA) at a rate of 500 Hz through 32 channels, as indicated by the grey ‘E’ discs in Figure 1A.

### 2.2. Data Preprocessing in EEGLab

The EEG data underwent comprehensive pre-processing and offline analysis using the EEGLab toolbox, an open-source software (https://sccn.ucsd.edu/eeglab/index.php accessed on 27 November 2023), designed for microstate analysis [18]. Initially, the data were downsampled to 250 Hz and high pass filtered at 1 Hz. To eliminate line noise, the ‘cleanline’ function was applied, followed by the ‘clean_rawdata’ function to identify and reject problematic channels. The interpolation of bad channels was accomplished using spherical splines [32] within the ‘clean_rawdata’ function, followed by re-referencing the EEG time series to the global average.

Task epochs were defined from the ‘start’ trigger by the experimenter, marking the initiation of the FLS task for each subject. The data then underwent artefact subspace reconstruction (ASR) using default settings in EEGLab, followed by re-referencing the EEG time series to the global average. ASR, an automated method, effectively removed transient EEG artefacts [33]. The default ASR parameter value of 20 was used, balancing the removal of non-brain signals with retaining brain activity, with the optimal range typically between 20 and 30 [33].

To focus on cortical sources corresponding with fNIRS HbO activity [20], a Laplacian spatial filter was applied to eliminate volume conduction from subcortical sources. Two expert subjects were excluded from the analysis due to the presence of ≥5 bad channels, as reliable microstate analysis requires the maximum number of bad channels to be less than five per subject [34]. Consequently, eight expert subjects remained in the study.

### 2.3. Data Processing of EEGLab and BCILab for Microstate-Based CSP Analysis

Microstate analysis was performed using the EEGlab toolbox [35] after aggregating EEG data during the FLS task from all experts (N = 8) and novices (N = 13), which is detailed in our published study [20]. In this study, we investigated the FLS complex task onset response where a previous study [36] demonstrated that the concentration of oxyhemoglobin peaked within 10 seconds during complex motor actions. Therefore, a 10 s duration was deemed sufficient for investigating the FLS complex task onset response using EEG and functional near-infrared spectroscopy [20]. FLS task related EEG dynamics will continue beyond the initial 10 seconds, which was not investigated in this study. First, we identified EEG microstate prototypes based on modified K-means clustering in EEGlab. The modified clustering of K-means was based on the goodness of fit of the microstate segmentation determined from the global explained variance (GEV) and the cross-validation criterion (CV). Here, the GEV criterion should theoretically become monotonically larger with increasing number of clusters [35]. The modified clustering of K-means in EEGlab found topographical maps of polarity-invariant microstate prototypes [35] from spontaneous EEG data during the FLS task (and the rest periods between the trials). Here, global field power (GFP) peaks are used to segment the EEG time series. The minimum peak distance was set at 10 ms (default) and 1000 randomly selected peaks (default) per subject were used for segmentation. Then, we rejected the GFP peaks that exceeded the standard deviation of all GFPs of all maps one time to segment the EEG data into a predefined number (2 to 8) of microstates. Here, the goal is to maximise the similarity between the EEG samples and the prototypes of the microstates they are assigned to using the modified K-means algorithm [35]. The modified K-means algorithm also sorts the microstate prototypes in decreasing GEV. We had set 100 random numbers of initialisations and 1000 maximum iterations for the modified K-means algorithm with 1 × 10^−6^ (default) as the relative threshold of convergence [35]. These microstates provided prototypes for subsequent microstate-based CSP analysis [21].

Microstate labels were applied to EEG samples from experts and novices based on topographical similarity (called backfitting) using the EEGlab toolbox [35]. Modified K-means algorithm [35] benefits of using k-means++ [37] for initialisation and the squared Euclidean metric for similarity calculation. Since short periods of unstable EEG topographies can occur, we applied temporal smoothing. Then, the temporally smoothed EEG topographies of experts (N = 8) and novices (N = 13) at the start of the FLS task in a 10 s time window were subjected to CSP analysis and classification using BCILab [38]. Here, if X_1_ and X_2_ are the EEG topographies from the experts and novices at the start of the FLS task, viz., X_1_ is a matrix of rows 250 Hz × 10 s (=2500 data points) and columns 32 channels, then, the desired spatial filter is obtained by, $\underset{w}{\mathrm{argmax}}J\left(w\right)=\frac{w^{T}X_{1}^{T}X_{1}w}{w^{T}X_{2}^{T}X_{2}w}=\frac{w^{T}C_{1}w}{w^{T}C_{2}w}$, where *w* denotes the spatial filter, and C_1_ and C_2_ represent the covariance matrices of X_1_ and X_2_, respectively. Using the Lagrange multiplier approach, the optimisation problem can be written as, $L\left(\lambda ,w\right)=w^{T}C_{1}w-\lambda \left(w^{T}C_{2}w\right)$, where λ is the Lagrange multiplier. The optimisation problem to find the spatial filter, *w*, requires the derivative set to zero, i.e., $\frac{\delta L}{\delta w}=2w^{T}C_{1}-2\lambda w^{T}C_{2}=0$. The solution to this optimisation problem are the eigenvectors, $M=C_{2}^{-1}C_{1},$ representing the spatial pattern vectors on the scalp. Here, the regularised CSP can improve robustness in small sample setting [39], and the largest eigenvector from, $M_{1}={\left(C_{2}+\alpha K\right)}^{-1}C_{1}$ and $M_{2}={\left(C_{1}+\alpha K\right)}^{-1}C_{2},\;$represent the spatial pattern vectors on the scalp with K assumed as an identity matrix [40]. Then, the classification was performed using a simple linear discriminant analysis (LDA) with a 10-fold cross-validation. The computational pipeline, starting from the raw EEG data to the classification, is shown in Figure 1B. Figure 1C shows the repeated measure design with 3 min for the FLS task and 2 min for the rest period.

### 2.4. Data Processing for Topography-Preserving CNN Approach

**Figure 2:**
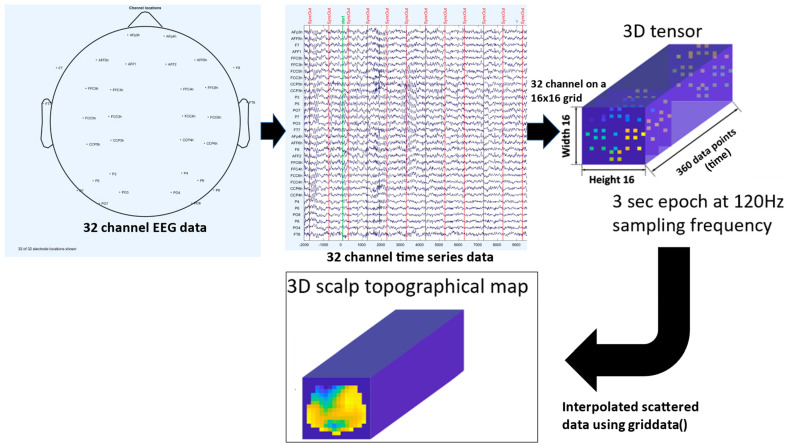
Transformation of 3 s of EEG time window into a 3D tensor for CNN analysis.

The procedure to convert the EEG data into a cuboid tensor is represented in Figure 2. Since spatiotemporal patterns were considered distinctive between experts and novices, spatiotemporal patterns were represented as 3D data that contained spatial information in two dimensions as well as temporal information in the third dimension. The EEG time series obtained from each channel was first downsampled at 120 Hz [28] and then projected to the corresponding position (from the scalp EEG montage; see Figure 1A) into a 2D image of a 16 × 16 square grid (height × width) using an azimuthal equidistant projection. We followed [28] so the EEG data during the FLS task from 2 s before the start trigger was divided into 3 s segments using a 1-second sliding window. The process was repeated in all sliding time steps, and the empty locations on the 16 × 16 grid between the projected electrode locations were interpolated using the griddata() function, Matlab (Mathworks, Inc., Natick, MA, USA) built-in function. Here, the griddata() function grids process 2D or 3D scattered data with a desired interpolation method. We used the v4 interpolation method in Matlab (Mathworks, Inc., USA) for better quality instead of cubic spline interpolation in ESNet [28]. Finally, the EEG data was shaped as a 3D tensor that included spatio-temporal information (that is, height × width × time). Here, we generated $X^{eeg}\in {\mathbb{R}}^{16\times 16\times 360}$ 3D EEG image for each 3-seconds (360 data points) EEG time window according to ESNet [28]. Therefore, 21 subjects (8 Experts and 13 Novices), with each subject performing the task three times (trials or reps), provided 21 subjects × 3 reps × 180 time windows (=11,340 cuboid tensors). 

### 2.5. CNN for the 3D-EEG Tensor Classification of Expert versus Novice

**Figure 3:**
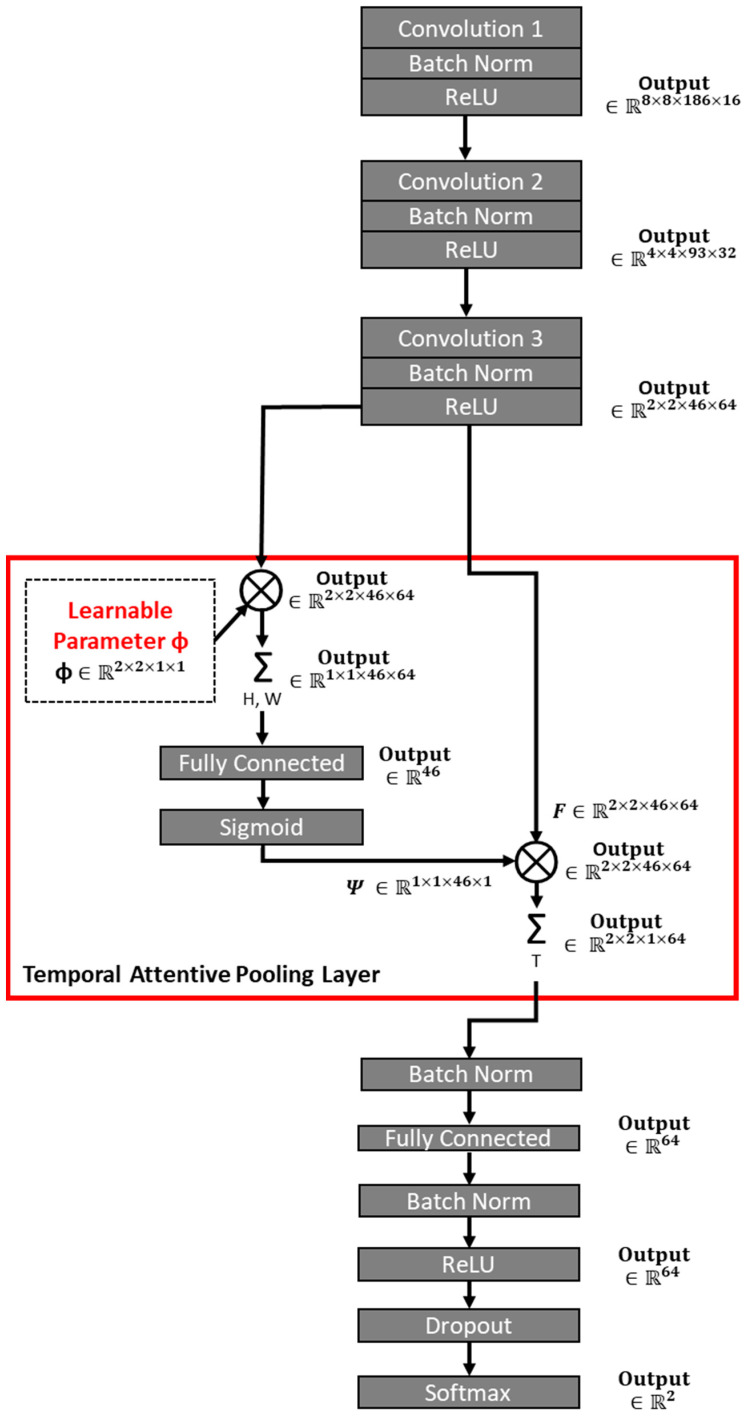
Our customised ESNet [28] architecture. The red box shows a temporal attentive pooling layer that was designed to compress temporal information extracted by consecutive convolutional layers efficiently. This pooling layer assigns higher weights to crucial time segments, enhancing the model’s focus on temporally significant features within the 3D feature representation.

A 3D CNN model, called ESNet [28], takes into account both spatial and temporal information by implementing a specific pooling layer called temporal attentive pooling (TAP) layer that compresses temporal information efficiently. The structure of the 3D CNN model is shown in Figure 3, which we adapted from ESNet [28]. The model consisted of three convolutional layers, and each of them is followed by a rectified linear unit (ReLU) activation function. Each layer inputs the channel information and doubles it as an output. Short-length kernels and strides were used for spatial information (i.e., the first and second axes) in each convolutional layer. Then, for temporal information (i.e., the third axis), short length kernels and stride were used in the second and third convolutional layers, while longer kernels and stride lengths were used in the first convolutional layer [28]. In summary, we determined the size of [kernel, stride] = [(2, 2, 10), (2, 2, 4)] for the first layer, [(2, 2, 2), (2, 2, 2)] for the second layer, and [(2, 2, 3), (2, 2, 2)] for the third layer. After the three convolutional layers, the TAP layer followed an efficient pooling process in the CNN model. The TAP layer first conducts Spatial Attentive Pooling (SAP), where the characteristic after the third convolutional layer is multiplied element by element by a trainable parameter, φ∈${\mathbb{R}}^{2\times 2\times 46\times 64}$, and then computes the sum along the spatial axis for the characteristic, resulting in the SAP feature shape of ${\mathbb{R}}^{1\times 1\times 46\times 64}$. Then, the SAP feature was classified by a Fully Connected (FC) layer, followed by the Softmax activation function, and multiplied element by the original feature after the third convolutional layer, and then, the sum along the temporal axis for the result was calculated. In total, the entire TAP process converted the shape of the original feature ${\mathbb{R}}^{2\times 2\times 46\times 64}$ to ${\mathbb{R}}^{2\times 2\times 1\times 64}$, providing a larger weight to the relevant temporal information and, therefore, compressing it efficiently. The detailed structure and concept of the TAP layer are described in the original paper [28]. Then, the feature after the TAP layer was passed through the FC layer, followed by the ReLU function, Dropout, and Softmax layer. In addition to the dropout layer, we further implemented the batch normalisation between each convolutional layer and the ReLU function, after the TAP layer and FC layer and the ReLU function. The L2 regularisation was also adapted in a kernel and bias at each FC layer with a regularisation factor of 0.01 to prevent overfitting. The mechanistic insights were based on Gradient-weighted Class Activation Mapping (Grad-CAM) for “visual explanations” of decisions from our CNN-based model [25]. GradCAM uses the gradients of the EEG map flowing into the final convolutional layer to produce a coarse localisation map highlighting the salient regions for expert versus novice classification.

### 2.6. CNN Classification & Evaluation Criteria

**Figure 4:**
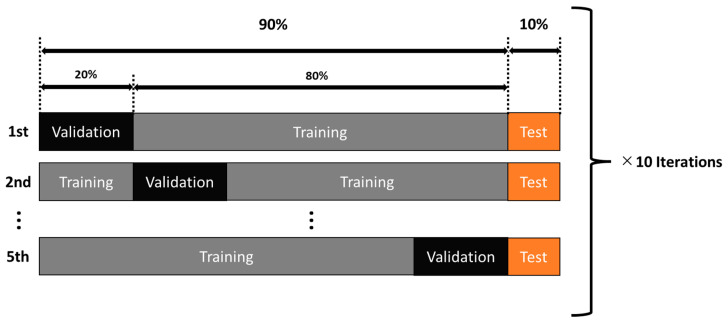
Data distribution for the five-fold cross-validation procedure that was repeated 10 times that generated a new training and validation splits of the trials, at random. Test data, shown in orange, remained the same for all the 10 repeats of the five-fold cross-validation.

We applied our customised ESNet [28], as shown in Figure 3, to classify experts versus novices with a five-fold cross-validation. In the five-fold cross-validation and testing, we divided the participants data in the 9:1 ratio, in which 10% of the total experts (8 subjects × 3 reps × 180 time windows) and novices (13 subjects × 3 reps × 180 time windows) data were used as holdout test data, and 90% of the total data were used for ten repeats of five-fold cross-validation. In each five-fold cross-validation, we trained the model using 80% of the 90% of total experts (8 subjects × 3 reps × 180 time windows) and novices (13 subjects × 3 reps × 180 time windows) data and cross-validated the model using 20% of the 90% total data, as shown in Figure 4. In each training epoch, the batch size was set at 32, and the training epoch was repeated 200 times, with five iterations within a five-fold cross-validation, with the learning rate set at 0.001. Then, for testing, this five-fold cross-validation was repeated 10 times that generated a new training and validation splits of the trials, at random, where we initialised the weights each time using the Keras initialiser function ‘glorot_uniform’, in which random values are pulled as initialised variables. The results of each iteration were evaluated with the holdout test data (10% of the total data) using indices: accuracy, F1 score, Mathews correlation coefficient (MCC), sensitivity, and specificity. The definition of F1 score, MCC, sensitivity, and specificity are as follows:

$$F1=\frac{\left(precision\right)\times \left(recall\right)}{\left(precision\right)+\left(recall\right)}\times 2\;\;$$

$$MCC=\frac{\left(TP\times TN-FP\times FN\right)}{\sqrt{\left(TP+FP\right)\left(TP+FN\right)\left(TN+FP\right)(TN+FN})}$$

$$Sensitivity=Recall=\frac{TP}{TP+FN}$$

$$Specificity=\frac{TN}{FP+TN}$$

Here, precision is shown as follows:

$$Precision=\frac{TP}{FP+FP}\;$$

Here, TP, FP, FN, and TN are the elements of the confusion matrix for binary classification.

C=[TP, FP; FN, TN] 

Moreover, TP (true positive) and FP (false positive) are the ratios of data correctly and falsely classified as positive data (i.e., Expert), respectively. Then, FN (false negative) and TN (true negative) are the numbers of data correctly and falsely classified as negative data (i.e., Novice), respectively.

## 3. Results

### 3.1. Microstate-Based CSP Classification of Expert versus Novice

**Figure 5:**
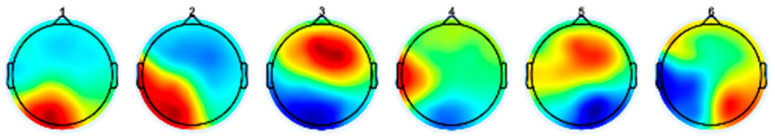
The 1–6 EEG microstate prototypes are sorted by decreasing global explained variance. These six EEG microstates are thought to represent basic building blocks of cognitive and perceptual processes during FLS skill learning [20].

We selected six microstate EEG prototypes based on the global explained variance (GEV) and the cross-validation criterion (CV) that was published earlier [20]. Here, the CV criterion, which points to the best clustering solution at its smallest value, reaches the minimum value for six microstates that are shown in Figure 5, sorted in decreasing GEV. As expected for a visuomotor task, the highest GEV is for microstate 1, corresponding to activation of the visual cortex (visual imagery [41]). The six microstate prototypes were backfitted to the EEG for 10 s at the start of the FLS task where it is postulated to be the start of building a cognitive-perceptual model [3,4] by the novices [6].

The global field power (GFP) of the first active microstates at the start of the FLS task was subjected to CSP analysis to find the spatial filters. Then, after applying spatial filters to the expert and novice EEG data and extracting features from them, the experts and novices were classified by LDA. We compared our microstate-based regularised CSP approach with conventional regularised CSP, where the microstate-based regularised CSP approach achieved a classification accuracy of 90.84% compared to 82.26% with conventional regularised CSP. The scalp topography for the first spatial filter using microstate based regularised CSP approach identified topographical maps from microstates 2 and 4 as the most significant eigenvectors. Also, our microstate based regularised CSP approach achieved classification accuracy greater than 90%. Here, microstate analysis applied a meta-criterion favouring the highest signal-to-noise ratio [24] that improved the accuracy when compared to that of conventional regularised CSP. Furthermore, microstates 2 and 4 as the most significant eigenvectors illustrated the importance of EEG electrodes in the parietal–temporal–occipital region for the classification of experts and novices during the FLS task. Microstate 2 was dominant in novices, while microstate 4 was dominant in the experts. We also computed the Kappa coefficient, which is a statistical method to measure the degree of agreement between classes. The Kappa coefficient method assigns zero to random classification and one to perfect classification [42], which is a more robust criterion than classification accuracy by considering random agreement. The microstate based regularised CSP approach outperformed conventional regularised CSP with a Kappa coefficient of more than 0.9. Importantly, the regularised CSP approach identified topographical maps from microstates 2 and 4 as the largest eigenvectors (from $M_{2}={\left(C_{1}+\alpha K\right)}^{-1}C_{2}$ and $M_{1}={\left(C_{2}+\alpha K\right)}^{-1},\;\mathrm{respectively}$).

### 3.2. CNN for EEG 3D Tensor Classification of Expert versus Novice

#### Five-Fold Cross-Validation

**Figure 6:**
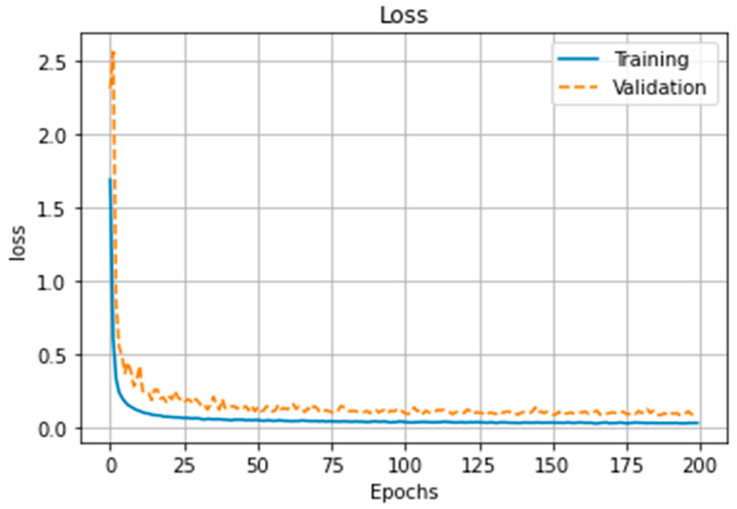
Training and validation loss function performance of the model.

**Figure 7:**
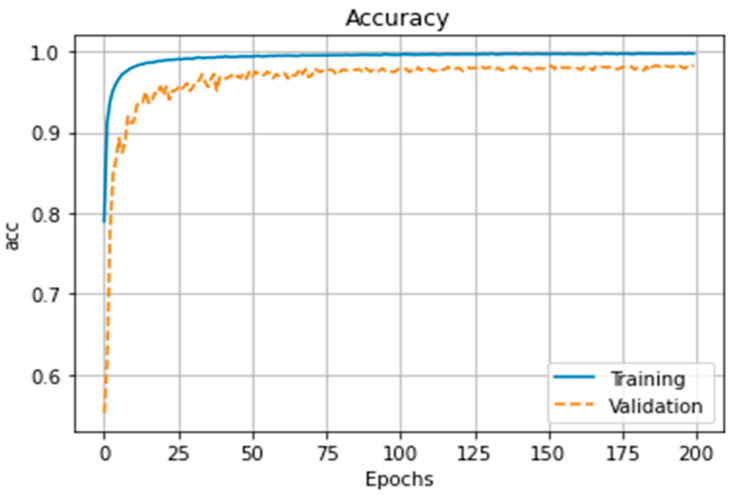
Accuracy performance of model training and validation.

Figure 6 and Figure 7 show the loss function and accuracy of the model, respectively, during 200 epochs of training and validation processes. The learning curve converges at the middle (100th epoch) of the training epochs, and the accuracy performance gap between training and validation stays within 2.5% by the end. Table 1 shows the average and maximum accuracies during the learning phase are shown for each of the 20 epochs.

### 3.3. Ten Evaluations with the Holdout Test Dataset

Table 2 shows the mean ± standard deviation and maximum for F1, MCC, precision, sensitivity, and specificity for the holdout test dataset over 10 repetitions of five-fold cross-validation. In Table 2, the highest values of each assessment (F1, MCC, accuracy, sensitivity, and specificity) are highlighted in red. Since our previous results of the classification accuracy using microstate-based CSP and LDA were 90.53%, so the topography-preserving CNN resulted is a significant improvement with >98% classification accuracy. The highest sensitivity, which indicates the percentage of correct predictions in the data labelled as positive (i.e., Expert), is 99.30% maximum. Then, the specificity, the rate of correct predictions in data labelled as negative (i.e., Novice), is 99.70% maximum. Furthermore, the F1 score, which evaluates the trade-off between recall and precision, reached 98.51%, indicating the equivalence of the model classification. Finally, the Matthews correlation coefficient (MCC), which evaluates the trade-off between the precision of positive and negative classifications (ranging from −100% to 100%), had a maximum of 97.56%. This implied that there was almost no classification bias among novices and experts even after five-fold cross-validation was repeated 10 times that generated a new training and validation splits of the trials, at random—see Table 2.

### 3.4. Gradient-Weighted Class Activation Mapping (Grad-CAM) Assessment of the CNN

**Figure 8:**
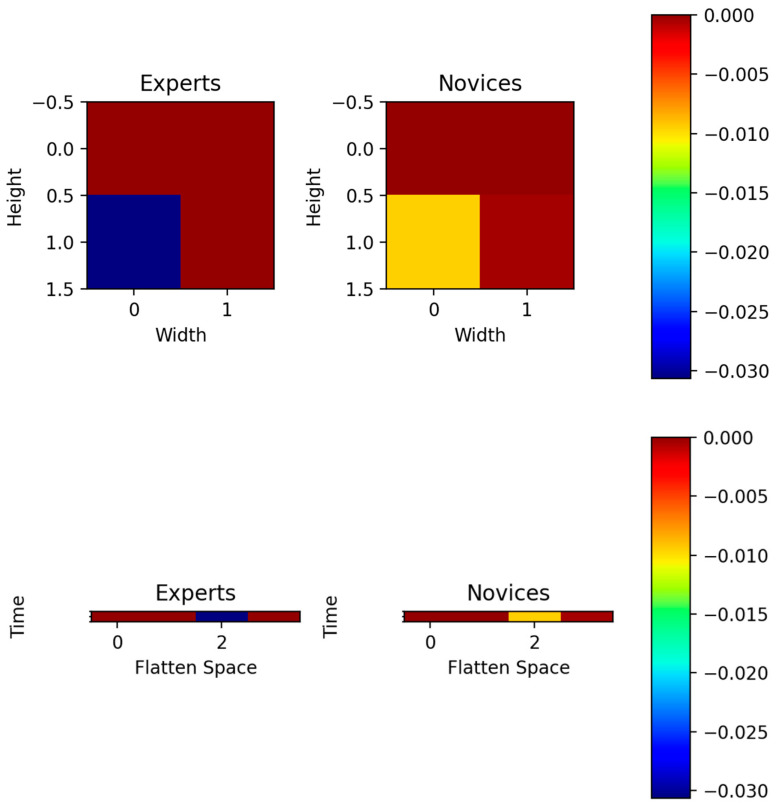
Spatial and temporal information are integrated through the incorporation of a dedicated pooling layer known as the temporal attentive pooling (TAP) layer, designed to efficiently condense temporal information. An illustrative Gradient-weighted Class Activation Mapping (Grad-CAM) assessment of TAP layer is shown where the top panel shows the compressed central tendency, while the bottom panel shows temporal activation (time in seconds) with flattened 2D grid (256 = 16 × 16) on the 1D x-axis.

The input of 16 × 16 EEG grid data to CNN (see Figure 3) is shown in the supplementary materials in Figure S1A for experts and novices. Then, the Grad-CAM heatmap for the convolutional layer 1 is shown in Figure S1B, for convolutional layer 2 is shown in Figure S1C, for the convolutional layer 3 is shown in Figure S1D, and for the TAP layer is shown in Figure 8. Note that the TAP layer first conducts Spatial Attentive Pooling which can provide insights into salient brain areas distinctive between experts and novices. The heatmap shows the salient regions in the topography-preserving convolutional layers from 1 to 3 (see Figure 3) where the time compressed central tendency of the heatmap for the TAP layer is shown in the top panel of Figure 8. The bottom left quadrant of the 16 × 16 EEG grid data is the discriminating salient region between experts and the novices. This bottom left quadrant is denoted by ‘2′ on the 1D x-axis in the bottom panel of Figure 8 showing temporal activation with flattened 2D space. The discriminating salient regions from the TAP layer corresponded (see E9, E10, E11, E12, E13, E14, and E15 in Figure 1A) with the centro-parietal (CCP5h, CCP3h), parietal (P3, P5, P7), and parieto–occipital (PO3, PO7) regions that partly overlap with the significantly different regions between experts and novices in our prior study [20].

## 4. Discussion

In this study, the novice trainees attempted a complex visuomotor task in a novel laparoscopic environment; therefore, they had to start building the perceptual model of the novel 3D environment based on 2D video and tactile feedback [43]. EEG topography, for example, microstate topographies, can be used as a marker of proficiency such that FLS psychomotor tasks with increasing task complexity can progress in the simulator as the novice trainee achieves proficiency towards FLS certification. For example, microstate 4 (see Figure 5) has been associated with the activation of the left inferior parietal lobe [44] related to the level of expert skill [45]. In our previous study [20], the microstate 4 was found to be more common in experts who are expected to have the action semantic knowledge [45]. Furthermore, global gestalt perception [46] is postulated to be present in experts due to their experience. Here, EEG topography can provide neurophysiological insights [20], e.g., microstate 1 (corresponding to visual cortex [41]), microstate 3 (corresponding to attention reorientation [41] and medial frontal cortex activation [47], and microstates 5 (topography comparable to microstate 3) during task performance can be considered markers of expertise. Then, the sequential flow of information between different brain states can be related to microstate sequences corresponding to the perception–action coupling [20]. 

We postulated that the CNN approach can learn the underlying temporal dynamics and provide latent representations that can be sensitive to other factors such as mental stress [26]. In this study, the ESNet approach [28] using EEG topography was adapted to classify experts from novices that provided a significant improvement with the highest sensitivity of 99.30% and the highest specificity at 99.70%. Since our CNN is topography-preserving, the Grad-CAM heatmap highlighting the bottom left quadrant of the TAP layer aligned with microstate 2 (see Figure 5) was found dominant in the novices from microstate-based CSP analysis. Here, microstate 2 is comparable to right-frontal left-posterior microstate A of the prototypical microstate classes [18,48], whereas microstate 4 hotspot that overlies the temporoparietal junction and the left inferior parietal lobe [44] may be related to the intact perception of global gestalt [49]. In particular, the Grad-CAM heatmap in the bottom panel of Figure 8D highlighted the parietal–occipital association area in novices (when compared to experts) at the beginning of the FLS task. This requires further investigation based on higher density EEG source localisation, since parietal hotspot was also found to be important for discrimination (relevant for spatial binding [46] based on CSP analysis which aligns well with the cognitive perception models [3,4]). Then, the supplementary motor area complex (SMA) is postulated to play a central role in the descent from the prefrontal to the motor cortex for the flow of skill-related information [7]. Here, SMA is known to be involved in planning complex motor finger tasks [50], and considered the programming area for motor effector subroutines in bimanual coordination tasks. Additionally, SMA has been suggested to form a queue of time-ordered motor commands before voluntary movement is executed via the descending pathways of the primary motor cortex (M1). In the current study, microstate 5 is postulated to capture SMA-related brain activity, which was found to be more frequent in experts than novices.

In the context of the perception–action cycle [51], investigation of motor skill acquisition with different virtual and physical simulation technologies [52] can provide insights into the neurophysiology of skill learning. Specifically, perception and action form a functional system through which behaviour is adapted in novices during exploratory actions to develop perceptual memory at the beginning in a novel environment. Then, perceptual memory allows action planning for improved skilled behaviour by updating the action parameters and refining them in executive memory, a continuous process of exploitative learning to reduce task variability. The two crucial attributes of the perception–action cycle are perceptual and executive memory [53], which are subserved by the frontoparietal network [54]. Here, the EEG-based analysis using microstate-based CSP and ESNet identified primarily the parietal–temporal–occipital EEG electrodes (microstates 2 and 4, the most significant eigenvectors) that illustrated the importance of the parietal–temporal–occipital association region for the classification of experts and novices. 

In our previous study [55], we found that average fNIRS HbO-based cortical activation in novices was mainly in the left pars opercularis of the inferior frontal gyrus involved in cognitive control [56]. The inferior frontal gyrus is postulated to be crucial for error-based learning [20] since published studies have shown that the inferior frontal gyrus and the presupplementary motor area (pre-SMA) are involved in stop signal task performance [57] that is relevant in error correction. Then, the prefrontal area [20,58] was found to be more active based on the activation of fNIRS HbO, which may be related to the manipulation of structured information [59]. Therefore, the fNIRS-guided attention network (FGANet) [28] may improve neurophysiological interpretation by capturing the frontoparietal hemodynamic network [54]. Specifically, neuroimaging of the rostrocaudal characteristics of the frontal lobes that are associated with varying degrees of information processing complexity [60] can be improved with fNIRS-EEG fusion where spatially important regions can be identified from fNIRS signals while temporally detailed neuronal activation, e.g., microstates, can be extracted from the EEG signals [20].

The main limitation of the current study is the low-density EEG montage since microstate analysis is more reliable with higher electrode densities [34,61,62]. Furthermore, a higher EEG electrode density can allow robust source localisation [63] to establish the regions of the brain underlying salient microstates that support skilled behaviour. Also, the limited number of subjects (8 experts and 13 novices) did not allow classification of individual skill level. Here, we conducted group comparison of EEG topography that may be too nonspecific to support clear conclusions about the skill level of individual subjects [64]. Therefore, the current study showed the feasibility of the CNN approach that substantially improved (>98%) EEG topography-based group classification of experts versus novices for FLS suturing with intracorporeal knot tying task when compared to microstate-based CSP analysis with LDA (~90%). Here, the accuracy performance gap between training and validation stayed within 2.5% by the end due to a limited number of subjects (see Figure 6 and Figure 7), and that gap did not lead to classification bias even after five-fold cross-validation was repeated 10 times and generated new training and validation splits, at random—see Table 2. A potential pitfall in using artifact removal using ICA in EEGlab is a decrease in rank that can cause decreased accuracy in the CSP implementation in BCIlab used in the current study [65]. Therefore, we have verified the spatial filters that they are not complex numbers in the current CSP analysis with LDA. Task onset trigger was set manually by the experimenter when the start command was assigned by him/her to the subject to start the FLS task, which can affect CSP analysis with LDA; however, the temporal attentive pooling layer of ESNet [28] can find temporally important time segments despite small (<10 s) misalignments. Then, a weakness of Grad-CAM used in this study is its partial derivative approach that can miss multiple occurrences of the same class and/or can lead to inaccurate localisation of a heatmap; therefore, Grad-CAM++ may be preferred in the future [66]. 

## 5. Conclusions and Future Research

We postulated that testing ESNet [28] for our application can provide mechanistic insights from EEG topography-preserving CNN approach that can be enhanced with a temporal attentive pooling layer using simultaneous fNIRS signals (see FGANet [28]). In the future research, FGANet [28] approach of online fNIRS-EEG fusion may drive closed-loop adaptive FLS simulators in virtual reality such that task difficulty may be individually paced according to brain-based metrics to develop “coping” to handle cognitive stress response (sympathetic vasoconstriction or ‘choking’ [67,68] monitored with portable neuroimaging [26]. Furthermore, subject-specific portable neuroimaging skill learning may provide brain-based error prediction [20] that can be compared with actual task errors from 3D (behaviour) video data (from FLS box trainer) to develop predictive fNIRS-EEG-video fusion. An expected task error can be highlighted in the 2D video feedback to novices to facilitate visuospatial attention for corrective action in the early stage of skill learning. Here, a distinction is necessary between sensory prediction error [69], which is postulated to be important at the initial perceptual-cognitive stage of skill learning [70], and task error which is postulated to be important in the later stages for strategy learning [9] to achieve expert performance. Then, the CNN with Grad-CAM approach provided insights into the main brain areas that differentiated experts from novices, which may be facilitated with neuroimaging-guided non-invasive brain stimulation—[58,71]. For example, non-invasive cerebellar stimulation may facilitate sensory prediction error and/or non-invasive frontal stimulation may facilitate task error feedback to improve FLS task performance and demonstrate brain-behaviour causality [72]. Also, a simultaneous multimodal EEG-fNIRS approach to measure task and/or non-invasive brain stimulation related brain response can provide important mechanistic insights, e.g., during non-invasive brain stimulation facilitated skill learning, where neurovascular coupling may be modulated by endogenous [73] and exogenous [74] arousals, e.g., due to sympathetic vasoconstriction [20,67,75].

## Figures and Tables

**Table 1:** Training and validation results: mean ± standard deviation and maximum accuracy during training and validation.

	Training Accuracy		Validation Accuracy	
Epoch	Mean ± Standard Deviation	Maximum	Mean ± Standard Deviation	Maximum
1	0.7895 ± 0.0157	0.8369	0.5522 ± 0.0398	0.7339
20	0.9883 ± 0.0016	0.9927	0.9570 ± 0.0140	0.9889
40	0.9934 ± 0.0013	0.9974	0.9685 ± 0.0099	0.9912
60	0.9952 ± 0.0008	0.9980	0.9699 ± 0.0126	0.9918
80	0.9956 ± 0.0011	0.9994	0.9709 ± 0.0105	0.9924
100	0.9962 ± 0.0010	0.9988	0.9779 ± 0.0080	0.9924
120	0.9966 ± 0.0009	0.9990	0.9796 ± 0.0062	0.9936
140	0.9973 ± 0.0008	0.9990	0.9794 ± 0.0070	0.9912
160	0.9972 ± 0.0008	0.9994	0.9793 ± 0.0065	0.9912
180	0.9971 ± 0.0008	0.9991	0.9778 ± 0.0096	0.9936
200	0.9975 ± 0.0008	0.9996	0.9836 ± 0.0038	0.9936

**Table 2:** Test results with the holdout test data (five-fold cross-validation repeated 10 times that generated a new training and validation splits of the trials): mean ± standard deviation and maximum for F1, MCC, precision, sensitivity, and specificity (confusion matrix for each iteration is shown in Table S1 in the supplementary materials). Numbers in **Bold** are highest across iterations.

Iteration	F1		MCC		Accuracy		Sensitivity		Specificity	
	Mean ± Standard Deviation	Maximum	Mean ± Standard Deviation	Maximum	Mean ± Standard Deviation	Maximum	Mean ± Standard Deviation	Maximum	Mean ± Standard Deviation	Maximum
1	0.9843 ± 0.0162	0.9928	**0.9756 ± 0.0221**	0.9887	0.9886 ± 0.0126	0.9947	**0.9930 ± 0.0197**	1.0000	0.9863 ± 0.0229	1.0000
2	**0.9851 ± 0.0171**	0.9913	0.9765 ± 0.0237	0.9864	**0.9891 ± 0.0138**	0.9937	0.9824 ± 0.0330	0.9942	0.9931 ± 0.0130	0.9983
3	0.9784 ± 0.0239	0.9869	0.9660 ± 0.0334	0.9796	0.9842 ± 0.0193	0.9905	0.9822 ± 0.0401	0.9971	0.9855 ± 0.0109	0.9882
4	0.9767 ± 0.0278	0.9871	0.9633 ± 0.0382	0.9796	0.9828 ± 0.0225	0.9905	0.9783 ± 0.0525	1.0000	0.9860 ± 0.0300	0.9950
5	0.9792 ± 0.0142	0.9843	0.9674 ± 0.0197	0.9754	0.9849 ± 0.0110	0.9884	0.9799 ± 0.0236	0.9940	0.9878 ± 0.0213	0.9983
6	0.9746 ± 0.0228	0.9843	0.9600 ± 0.0304	0.9752	0.9813 ± 0.0174	0.9884	0.9707 ± 0.0350	0.9908	0.9880 ± 0.0333	0.9967
7	0.9821 ± 0.0144	0.9871	0.9719 ± 0.0193	0.9796	0.9870 ± 0.0110	0.9905	0.9861 ± 0.0192	0.9940	0.9876 ± 0.0223	0.9950
8	0.9745 ± 0.0403	0.9942	0.9594 ± 0.0552	0.9909	0.9804 ± 0.0335	0.9958	0.9550 ± 0.0692	0.9942	**0.9970 ± 0.0117**	1.0000
9	0.9829 ± 0.0167	0.9871	0.9730 ± 0.0232	0.9798	0.9874 ± 0.0136	0.9905	0.9758 ± 0.0340	0.9912	0.9944 ± 0.0139	0.9983
10	0.9758 ± 0.0366	0.9899	0.9618 ± 0.0491	0.9841	0.9821 ± 0.0284	0.9926	0.9741 ± 0.0425	0.9912	0.9874 ± 0.0402	1.0000

## Data Availability

Data available on request due to privacy/ethical restrictions.

## Supplementary Materials

The following supporting information can be downloaded at: https://www.mdpi.com/article/10.3390/brainsci13121706/s1.
