# Supplementary material for: Distinguishing Laparoscopic Surgery Experts from Novices Using EEG Topographic Features
Source: Brain Sci. 2023 Dec 11;13(12):1706. doi: 10.3390/brainsci13121706 (PMC10742221; doi:10.3390/brainsci13121706)
Supplement: Supplementary file 1 [file brainsci-13-01706-s001.zip › brainsci-2725049-supplementary.pdf]

Table S1. Confusion Matrix for each iteration.

Ite	Val	Confusion Matrix				Ite	Val	Confusion Matrix			
		TP	FP	FN	TN			TP	FP	FN	TN
1	1	346	0	5	600	6	1	343	3	15	590
	2	334	12	1	604		2	323	23	3	602
	3	339	7	5	600		3	344	2	9	596
	4	336	10	0	605		4	344	2	17	588
	5	333	13	1	604		5	339	7	8	597
2	1	345	1	15	590	7	1	336	10	3	602
	2	342	4	2	603		2	331	15	2	603
	3	343	3	4	601		3	339	7	5	600
	4	339	7	4	601		4	343	3	6	599
	5	340	6	6	599		5	343	3	8	597
3	1	337	9	1	604	8	1	346	0	36	569
	2	334	12	7	598		2	341	5	3	602
	3	339	7	17	588		3	344	2	2	603
	4	338	8	5	600		4	344	2	14	591
	5	338	8	1	604		5	346	0	29	576
4	1	328	18	0	605	9	1	341	5	4	601
	2	330	16	2	603		2	339	7	3	602
	3	343	3	25	580		3	343	3	15	590
	4	343	3	6	599		4	345	1	8	597
	5	343	3	6	599		5	345	1	13	592
5	1	335	11	8	597	10	1	339	7	3	602
	2	337	9	6	599		2	345	1	12	593
	3	334	12	2	603		3	318	28	6	599
	4	342	4	9	596		4	346	0	21	584
	5	345	1	10	595		5	343	3	4	601
